# Corticosterone induces DNA methyltransferases expression in rat cortical neurons

**DOI:** 10.1186/2193-1801-4-S1-P50

**Published:** 2015-06-12

**Authors:** Mari Urb, Kaili Anier, Terje Matsalu, Anu Aonurm-Helm, Tõnis Timmusk

**Affiliations:** University of Tartu, Estonia; Tallinn University of Technology, Estonia

**Keywords:** DNA methyltransferase, maternal separation, corticosterone

Corticosterone (CORT) is the main glucocorticoid hormone involved in stress responses in rodents. It is established that CORT exerts its effects via glucocorticoid receptor (GR) and mineralocorticoid receptor that regulate downstream gene expression during development and adulthood. In our previous study, we have shown that maternal separation on postnatal day 15 increases DNA methyltransferase (DNMT) 1, 3A and 3B expression levels in rat nucleus accumbens lasting into adulthood (Anier et al. 2014). However, the exact mechanism how maternal separation alters DNMT expression is unclear. We hypothesize that stress-induced GR stimulation may increase the expression levels of DNMTs and alter long-term DNA methylation-demethylation balance in infant rat brain. Our aim is to evaluate the effect of CORT and maternal separation on the expression levels of DNMTs in rat cortex. In rat primary cortical neurons, CORT treatment increased mRNA levels of DNMT3A and DNMT3B. GR antagonist mifepristone significantly decreased CORT-induced DNMTs mRNA levels indicating GR stimulation-dependent upregulation of DNMTs expression. Higher mRNA levels of DNMT1, DNMT3A and DNMT3B in rat cortex at postnatal day 15 and increased plasma CORT levels suggest that elevated CORT upregulates DNMTs expression. Our results indicated that DNMTs are downstream targets of GR-dependent CORT stimulation and early life stress may induce aberrant DNA methylation pattern that could facilitate long-term changes in gene expression.
